# Generality of toxins in defensive symbiosis: Ribosome-inactivating proteins and defense against parasitic wasps in *Drosophila*

**DOI:** 10.1371/journal.ppat.1006431

**Published:** 2017-07-06

**Authors:** Matthew J. Ballinger, Steve J. Perlman

**Affiliations:** 1 Department of Biology, University of Victoria, Victoria, BC, Canada; 2 Integrated Microbial Biodiversity Program, Canadian Institute for Advanced Research, Toronto, ON, Canada; University of Liverpool, UNITED KINGDOM

## Abstract

While it has become increasingly clear that multicellular organisms often harbor microbial symbionts that protect their hosts against natural enemies, the mechanistic underpinnings underlying most defensive symbioses are largely unknown. *Spiroplasma* bacteria are widespread associates of terrestrial arthropods, and include strains that protect diverse *Drosophila* flies against parasitic wasps and nematodes. Recent work implicated a ribosome-inactivating protein (RIP) encoded by *Spiroplasma*, and related to Shiga-like toxins in enterohemorrhagic *Escherichia coli*, in defense against a virulent parasitic nematode in the woodland fly, *Drosophila neotestacea*. Here we test the generality of RIP-mediated protection by examining whether *Spiroplasma* RIPs also play a role in wasp protection, in *D*. *melanogaster* and *D*. *neotestacea*. We find strong evidence for a major role of RIPs, with ribosomal RNA (rRNA) from the larval endoparasitic wasps, *Leptopilina heterotoma* and *Leptopilina boulardi*, exhibiting the hallmarks of RIP activity. In *Spiroplasma*-containing hosts, parasitic wasp ribosomes show abundant site-specific depurination in the α-sarcin/ricin loop of the 28S rRNA, with depurination occurring soon after wasp eggs hatch inside fly larvae. Interestingly, we found that the pupal ectoparasitic wasp, *Pachycrepoideus vindemmiae*, escapes protection by *Spiroplasma*, and its ribosomes do not show high levels of depurination. We also show that fly ribosomes show little evidence of targeting by RIPs. Finally, we find that the genome of *D*. *neotestacea’s* defensive *Spiroplasma* encodes a diverse repertoire of RIP genes, which are differ in abundance. This work suggests that specificity of defensive symbionts against different natural enemies may be driven by the evolution of toxin repertoires, and that toxin diversity may play a role in shaping host-symbiont-enemy interactions.

## Author summary

Nearly all insects harbor bacterial partners. These microbes can be defensive symbionts, protecting their hosts against parasites and pathogens, and in some cases, may defend against more than one enemy, presenting opportunity to study the evolution of specificity underlying defensive interactions. What factors determine specificity and generality of defense? Can symbiont-encoded effector molecules act generally against enemies without causing harm to the host? We show here that symbiont-encoded ribosome-inactivating toxins, previously implicated in protection of a *Drosophila* fruit fly against its nematode parasite, are also implicated in defending flies against parasitic wasps. We quantify activity of the toxin as the proportion of ribosomes depurinated and, importantly, find that susceptible wasps are attacked early in development and are strongly affected, while hosts and a resistant wasp are not. We also show that not one, but a family of toxins is maintained and expressed by symbionts. Together, our findings implicate toxin diversity as a factor contributing to the evolution of specificity in a symbiont-mediated defense.

## Introduction

Multicellular organisms commonly harbor microbial symbionts. Some of the best-studied recent examples lie in maternally transmitted bacterial endosymbionts of insects, which are ubiquitous [1–3], and rely on the successful reproduction of their hosts to ensure their own survival. Unsurprisingly, this puts them in direct conflict with their hosts' natural enemies, and recent work has documented extraordinary diversity in insect symbiont-mediated protection. For example, native inherited bacterial endosymbionts confer resistance to parasitic wasps in aphids and *Drosophila* [4,5], predatory spiders in rove beetles [6], parasitic nematodes and RNA viruses in *Drosophila* flies [7–9] and pathogenic fungi in beewolves and aphids [10–12]. While an increasing number of examples are being uncovered, very little is known about the mechanism of symbiont-mediated protection. How specific is protection? Can symbionts use the same strategies to protect against different classes of natural enemies? How do symbionts recognize and target enemies without harming their host in turn?

In this study, we address the mechanism and generality of protection conferred by *Spiroplasma* defensive symbionts of *Drosophila* flies. *Spiroplasma* (Mollicutes) is a diverse and widespread lineage of arthropod-associated bacteria [13] thought to infect at least ~7% of insects [3]. *Spiroplasma* biology is incredibly diverse. While many strains are commensal in arthropod guts, a number of pathogenic strains have been identified, including pathogens of bees (*S*. *melliferum* and *S*. *apis*), crayfish (*S*. *eriocheiris*), and plants (*S*. *citri* and *S*. *kunkelii*) [14–17]. Plant-pathogenic strains are vectored by plant-feeding Hemipteran insects. In addition, vertical transmission has evolved independently in *Spiroplasma* numerous times, including in *Drosophila* flies, pea aphids, and butterflies [18–20]. Vertically transmitted *Spiroplasma* have evolved a number of interesting strategies to persist in their hosts, including male-killing and protection against natural enemies. Perhaps the best-studied defensive *Spiroplasma* infects the hemolymph and ovarian tissues of the woodland fly *Drosophila neotestacea*, which it protects against a common and virulent parasitic nematode, *Howardula aoronymphium* [9]. Indeed, the benefits of *Spiroplasma* protection are so great, and nematode parasitism is so prevalent in nature, that *Spiroplasma*-infected flies have replaced uninfected ones, with the symbiosis currently spreading across North America [21]. The *Spiroplasma* symbiont of *D*. *neotestacea* (hereafter *s*Neo) and the closely-related strains infecting *Drosophila melanogaster* (hereafter *s*Mel) and *Drosophila hydei* have also been shown to defend against parasitic wasps in the lab [5,22–25]. *s*Mel is also known as the melanogaster sex ratio organism, or MSRO, because it also acts as a male-killer [26], eliminating all sons of infected females during embryogenesis by targeting male-specific components of the dosage compensation complex [27–29]. *Spiroplasma* is highly effective in protecting against wasps in the lab, though not always in rescuing flies. Despite high rates of fly mortality in some cases, wasp killing results in a net benefit to *Spiroplasma*-infected hosts [22]. Parasitic wasps are among the most important sources of mortality against *Drosophila* in the wild [30,31], although whether *Spiroplasma* protection against wasps occurs in nature has not yet been demonstrated. Interestingly, unlike protection against wasps, which appears to be quite general to *Spiroplasma* in *Drosophila*, symbiont transfection experiments showed that only *s*Neo is able to protect against nematodes [23].

We recently made progress toward resolving how *Spiroplasma* protects *D*. *neotestacea* against parasitic nematodes. We found that *Spiroplasma* encodes a ribosome-inactivating protein (RIP) that is implicated in nematode defense [32]. RIPs are N-glycosidases that irreversibly inactivate eukaryotic cytosolic ribosomes by cleaving a specific adenine residue from the 28S ribosomal RNA (rRNA). Well-known RIPs include potent toxins such as ricin and Shiga toxin, both of which are categorized as type 2 RIPs. These RIPs are composed of an A-chain, a polypeptide which possesses N-glycosidase activity, bonded by disulphide linkages to a B-chain, a lectin or carbohydrate binding domain that is involved in attachment to the cell surface. RIPs possessing only an A-chain are categorized as type 1 RIPs; this type was previously found in *s*Neo. These may be equally effective ribosome inactivators *in vitro*, but are often less toxic *in vivo*, likely because cell entry is restricted.

The best studied RIPs are those found in angiosperms, where they are extremely diverse and common, and appear to act in defensive roles against both predators and viruses [33]. In addition to inhibition of protein synthesis, both type 1 and type 2 RIPs lead to apoptosis and necrosis in animals [34]. Bacterially-encoded RIPs have been much less studied, other than those found in *Shigella dysenteriae* and enterohemorrhagic *Escherichia coli* serotypes, which are notorious for their contribution to bacterial virulence in humans [35,36]. RIP homologs have been found in many bacterial genomes but few have been characterized. A gene encoding a Shiga-like toxin B-chain is among several toxin genes associated with protective strains of APSE, the bacteriophage harboured by *Hamiltonella defensa*, a defensive symbiont of aphids that confers protection against parasitic wasps [37–39], but this gene has not yet been functionally characterized.

We previously found that a type 1 RIP in *s*Neo is transcriptionally upregulated in nematode-infected flies [32], and attacks nematode ribosomes *in vitro* [40]. Furthermore, infection experiments revealed that nematodes that infect flies harbouring *Spiroplasma* exhibit the hallmarks of attack by RIP toxins, including massive increases in levels of depurinated ribosomes [40]. Intriguingly, the genome of the *Spiroplasma* symbiont of *D*. *melanogaster* contains five putative RIPs [41], hinting that RIP toxins also play a role in protection against parasitic wasps, and that successful protection against a specific enemy might depend on the specific arsenal of toxins encoded by each symbiont. We therefore explored the role of RIPs in parasitic wasp defense, exposing *Spiroplasma*-positive and negative flies to three wasp species, quantifying RIP toxin expression, as well as intact and depurinated ribosomes in wasps and hosts, and surveying the genome of *Spiroplasma* in *D*. *neotestacea* for additional RIPs.

We find strong evidence implicating *Spiroplasma*-encoded RIPs in protection against parasitic wasps. Wasp ribosomes are depurinated as soon as eggs hatch inside the fly larva, with depurination peaking upon host pupation. We find little evidence that fly ribosomes suffer significant collateral damage from RIPs. We also report the discovery of an ectoparasitic wasp that is not killed by *Spiroplasma* and escapes the brunt of RIP attack. Furthermore, we identify three additional putative RIP genes in the genome of *s*Neo, which protects against both nematodes and wasps, and show that this symbiont encodes two RIPs with no apparent close relatives in the *s*Mel genome. Our results contribute to a growing appreciation for the potential of symbiont-encoded toxins as important determinants of specificity in insect defensive symbioses.

## Results

### Parasitic wasp ribosomes show signatures of attack by *Spiroplasma* RIPs

**Fig 1:**
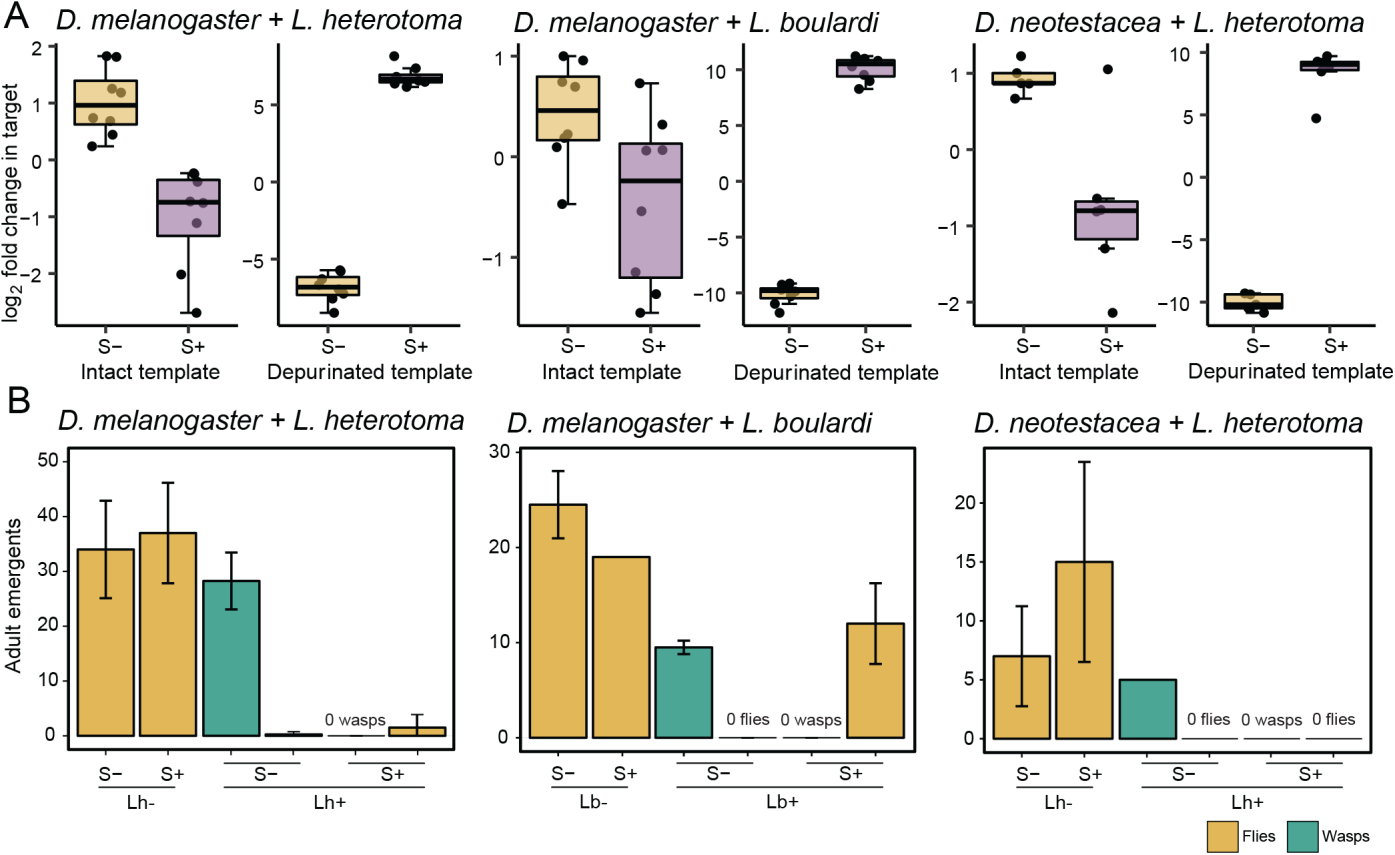
Parasitic wasp ribosomes are depurinated during host protection. Levels of intact and depurinated wasp ribosomes are significantly decreased and increased, respectively, in *Spiroplasma*-positive flies, compared to *Spiroplasma*-negative flies (A). Results of t-tests for intact ribosomes: *D*. *melanogaster* + *L*. *heterotoma*, *p* < 0.001; *D*. *melanogaster* + *L*. *boulardi*, *p* = 0.028; *D*. *neotestacea* + *L*. *heterotoma*, *p* = 0.010, and for depurinated ribosomes, all fly-wasp pairs, *p* < 0.001; n = 8, 8, 6, and 5 flies per treatment, for *D*. *melanogaster S+*, S-, and *D*. *neotestacea S+*, S-, respectively. Jitter points are the mean of two technical replicates per wasp-infested larva. Outcomes of wasp exposure for fly and wasp survival were measured as emerged adults (B). Each strain of *Spiroplasma* kills *Leptopilina*. *L*. *heterotoma* and *L*. *boulardi* emergence is significantly reduced in *Spiroplasma*-positive flies relative to *Spiroplasma*-negative controls (t-tests, *L*. *heterotoma* in *D*. *melanogaster*, *p* < .001; *L*. *boulardi* in *D*. *melanogaster*, *p* = .003; *L*. *heterotoma* in *D*. *neotestacea*, *p* < .001). Y-axes are labeled with total number of adult emergents per dish and error bars show the standard deviation of the mean number of adult emergents across replicate dishes (sample sizes: *L*. *heterotoma* in *D*. *melanogaster*, 60 larvae, 4 dishes; *L*. *boulardi* in *D*. *melanogaster*, 40 larvae, 2 dishes; *L*. *heterotoma* in *D*. *neotestacea*, 40 larvae, 2 vials).

To assess RIP activity on parasitic wasp 28S rRNA, we modified an established, highly-sensitive RT-qPCR-based assay [40,42,43] to quantify the abundance of ribosomes that have been depurinated at the α-sarcin/ricin loop (SRL), relative to the total ribosome pool. In separate reactions, primers specific to either intact or depurinated SRLs were used to quantify ribosomal targets in each state. The forward primers differ in sequence only at their 3’ termini, with one set of primers designed to hybridize with “intact” ribosomal cDNA synthesized from rRNA possessing an adenine at the affected site (adenine SRLs, thymine variant cDNA), and the other primer set targeting cDNA synthesized from rRNA containing the abasic position (depurinated SRLs, adenine variant cDNA). Strong specificity to wasp and fly ribosomes is conferred by the sequence of the reverse primer, which hybridizes with a less-conserved region nearby and prevents mis-amplification across primer sets. We found a remarkably strong signal of RIP attack, with elevated abundances of depurinated wasp ribosomes associated with *Spiroplasma*-positive flies (Fig 1A). We tested parasitized hosts on the first day of pupation and found high levels of depurination (t-test: *L*. *heterotoma* in *D*. *melanogaster* t_14_ = 34.17, *p* < .001, a >13,000-fold increase). On the second day of pupation, the extent of RIP attack was even more striking (t-tests: *L*. *heterotoma* in *D*. *melanogaster* t_10_ = 30.07, *p* < .001, a >800,000-fold increase; *L*. *boulardi* in *D*. *melanogaster* t_14_ = 41.21, *p* < .001, a >1,000,000-fold increase; *L*. *heterotoma* in *D*. *neotestacea* t_9_ = 21.09, *p* < .001, a >350,000-fold increase). These fold changes are all at the upper limit of our assay’s quantification sensitivity. The unambiguous signal of depurinated wasp ribosomes in *Spiroplasma*-positive hosts was accompanied by a strong reduction in the pool of intact wasp ribosomes in all symbiont-wasp encounters (Fig 1A, t-tests: *L*. *heterotoma* in *D*. *melanogaster* t_14_ = 5.39, *p* < .001, a 4.1-fold decrease; *L*. *boulardi* in *D*. *melanogaster* t_14_ = 2.45, *p* = .028, a 1.7-fold decrease; Welch’s t-test: *L*. *heterotoma* in *D*. *neotestacea* t_5.47_ = 3.88, *p* = .010, a 3.2-fold decrease). This pattern parallels our protection assays, in which we found almost complete wasp mortality in *Spiroplasma*-positive flies (Fig 1B), as has been previously shown [22,23]. Despite wasp death, flies parasitized by *L*. *heterotoma* (but not *L*. *boulardi*) are not rescued by *Spiroplasma*, as has also been previously shown [25]. These experiments clearly demonstrate that an attack involving ribosome depurination is mounted against parasitic wasps by *Spiroplasma* during host defense. The attack is followed by complete wasp mortality but differential host survival, possibly contingent upon the wasp species.

### The onset of RIP activity coincides with timing of wasp hatching

**Fig 2:**
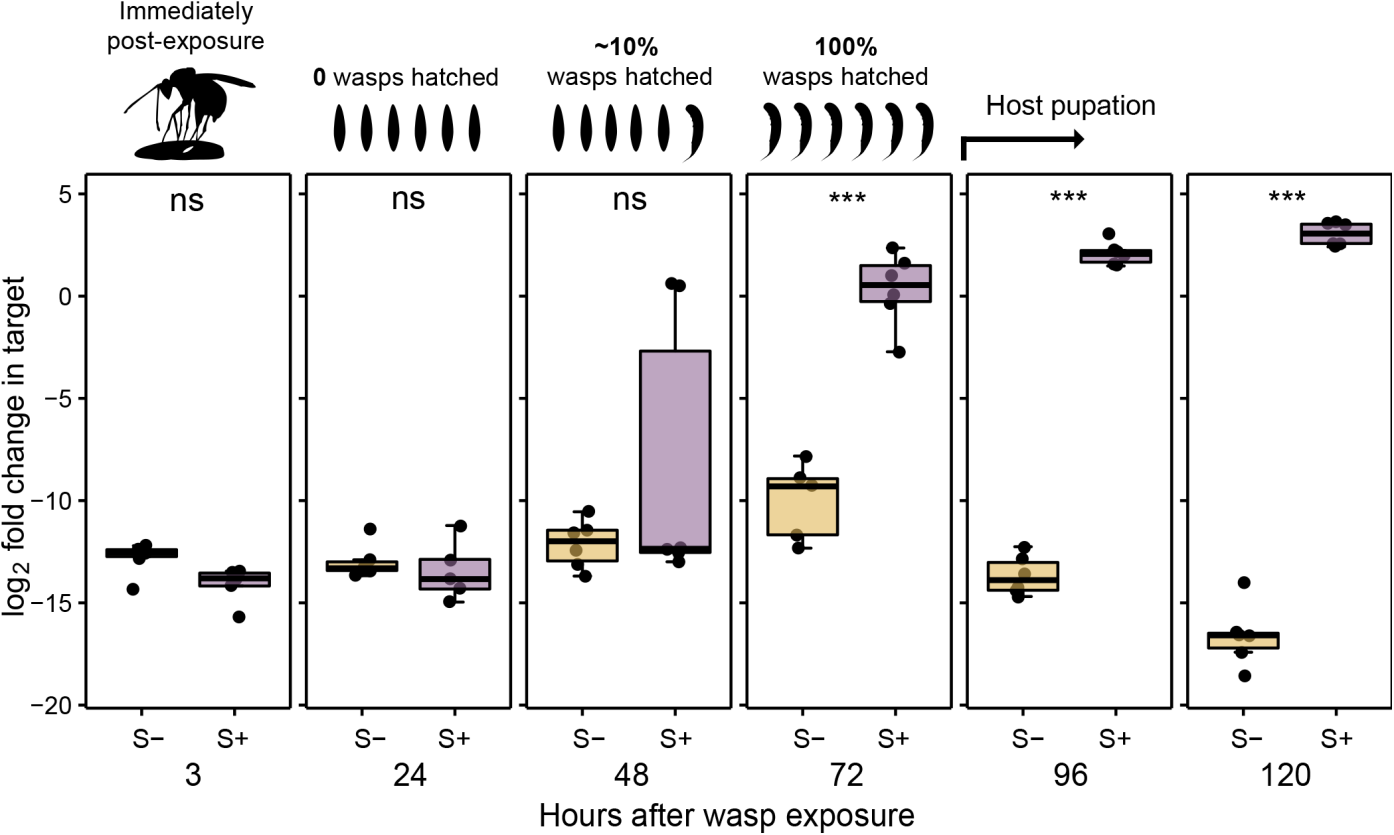
Hallmark of RIP attack in wasps begins soon after hatching. Abundance of depurinated ribosomes across a timecourse of *L*. *heterotoma* infestation of *D*. *melanogaster* in the presence and absence of *s*Mel (n = 6 fly larvae or pupae per timepoint). Jitter points are the mean of two technical replicates per wasp-infested larva. Depurinated *L*. *heterotoma* ribosomes are detected 48 hours after oviposition in *s*Mel-positive *D*. *melanogaster*, with increasing levels at subsequent timepoints. For all significant comparisons, *p* < .001 (t-tests). Legends above boxplots indicate parasitic wasp developmental stage at each time point indicated.

In order to determine the onset of depurination of wasp ribosomes, we carried out a detailed timecourse experiment, assaying wasp ribosomes for RIP activity immediately after exposing *D*. *melanogaster* to *L*. *heterotoma* and at subsequent 24-hour intervals until the second day of pupation (120 hours post-exposure). Our results reveal evidence of RIP attack remarkably early, just 48 hours after wasp exposure, with two of the six parasitized fly larvae tested showing a >3,000-fold increase in depurinated wasp ribosomes relative to *Spiroplasma*-free, *L*. *heterotoma*-infested controls (Fig 2; t_6_ = 13.2, *p* < .001). In the remaining four larvae, there was no significant change relative to *Spiroplasma*-free controls, suggesting RIP attack had not yet begun (t_8_ = 0.08, *p* = .933). All six fly larvae tested at the following time point, 72 hours after wasp exposure, showed a strong signal of wasp ribosome depurination (t_9_ = 9.56, *p* < .001).

The result at 48 hours led us to suspect that the onset of RIP activity might coincide with hatching of the wasp egg. Therefore, we carried out an additional exposure, identical in design to the previous, and dissected parasitized larvae at 48 hours (N = 20) and 72 hours (N = 11), scoring the frequency of unhatched eggs and hatched larvae at each time point. In our assays, wasps begin hatching around 48 hours after infestation. At this time, 16 fly larvae contained at least one wasp egg, just two contained a wasp larva (one of these contained both an egg and a larva), and three were unparasitized. In our 72 hour dissections, nine flies contained wasp larvae, two were unparasitized, and none contained wasp eggs.

Together, the results of these experiments suggest that RIP attack begins very early in wasp development, apparently as soon as newly hatched wasp larvae are exposed to host hemolymph. The early signal of depurination fits nicely with previous work showing wasp developmental delays in *Spiroplasma*-positive flies as early as 72 hours after infestation [22].

### ‘Collateral damage’ to host ribosomes?

**Fig 3:**
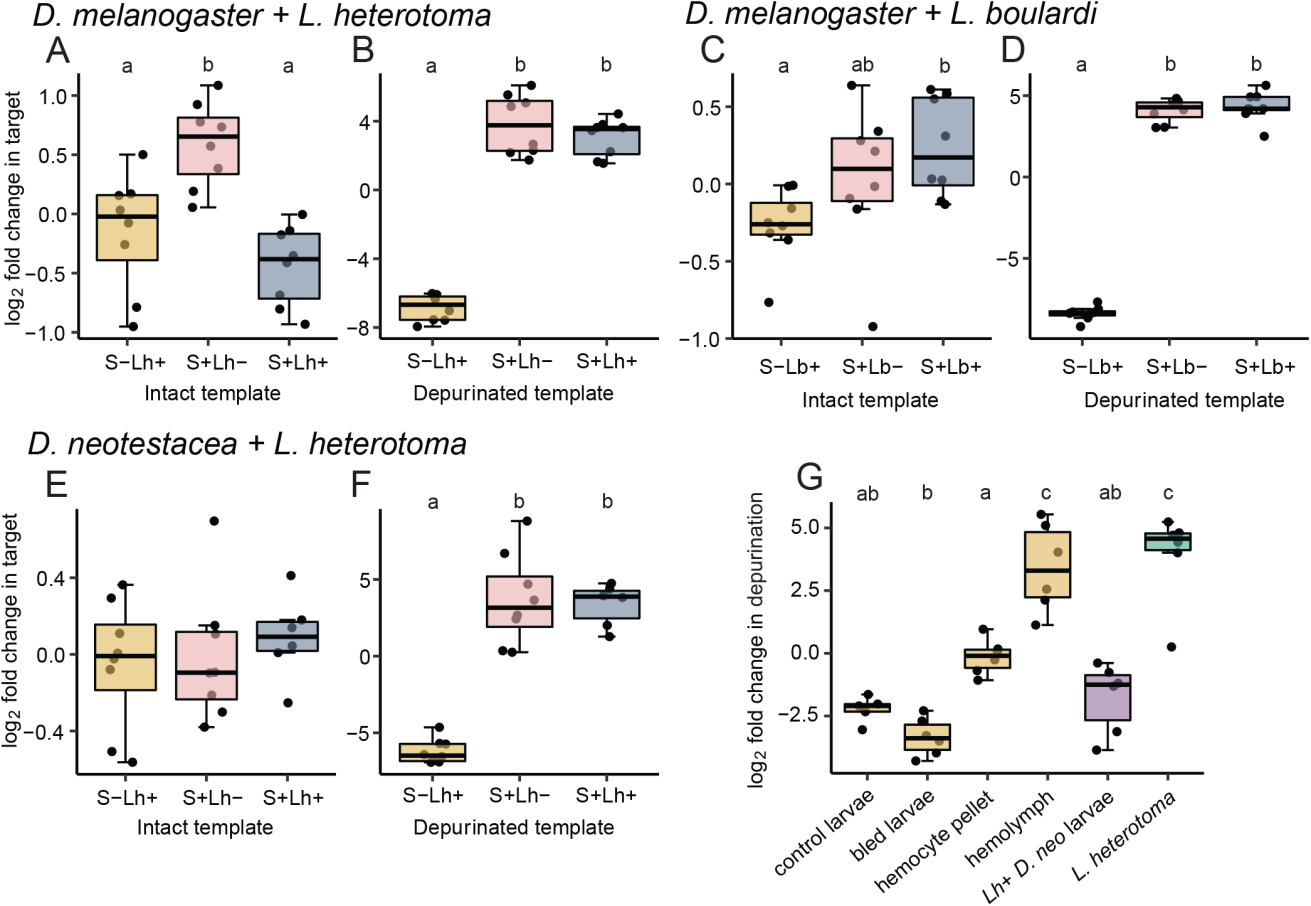
Depurination of *Drosophila* ribosomes is minimal and does not explain host mortality during parasitic wasp infestation. Levels of intact *Drosophila* ribosomes are not significantly decreased in *Spiroplasma*-positive flies (blue), compared to *Spiroplasma*-negative flies (gold) (A,C,E; *p* = .348, *p* = .026, *p* = .693 for *L*. *heterotoma*-infested *D*. *melanogaster*, *L*. *boulardi*-infested *D*. *melanogaster* and *L*. *heterotoma*-infested *D*. *neotestacea*, respectively). Depurinated *Drosophila* ribosomes are significantly more abundant in *Spiroplasma*-positive (blue) compared to *Spiroplasma*-negative flies (gold; B,D,F; *p* < .001); however, there is no difference in abundance of depurinated *Drosophila* ribosomes between wasp-infested (blue) and uninfested hosts (pink) regardless of fly survival outcome following defense. Jitter points are the mean of two technical replicates per larva. (G) Levels of depurinated *Drosophila* ribosomes are plotted for samples of host hemolymph, hemocytes, bled larvae and unbled controls. Jitter points are the mean of two technical replicates per pool of eight larvae. The proportion of depurinated host and infesting wasp ribosomes from larval samples collected in a separate experiment are shown for comparison. Post hoc significance test results are shown above boxplots (Tukey tests).

A major question in defensive symbiosis is whether and how symbionts distinguish hosts from natural enemies, and whether hosts exhibit any delayed deleterious effects of symbiont-mediated protection. Specifically, we hypothesized that host mortality in *L*. *heterotoma*-exposed, *Spiroplasma*-positive flies might be due to off-target RIP attack. To address this, we quantified intact and depurinated host ribosomes, using the same parasitized flies that we used in our wasp ribosome assays. Although we were able to detect significant levels of depurinated host ribosomes relative to *Spiroplasma*-negative controls (Fig 3B, 3D and 3F, ANOVAs: *L*. *heterotoma*-infested *D*. *melanogaster*, *F*_2,21_ = 176, *p* < .001, a ~950-fold increase; *L*. *boulardi*-infested *D*. *melanogaster*, *F*_2,21_ = 826, *p* < .001, a > 6,000-fold increase; *L*. *heterotoma*-infested *D*. *neotestacea*, *F*_2,19_ = 60.7, *p* < .001, a ~750-fold increase), their proportion relative to total ribosomes was much lower than in wasps and was not elevated in wasp-infested relative to uninfested flies, suggesting that RIP activity is not responsible for host mortality (Fig 3B, 3D and 3F, pink versus blue boxes; plots are labeled with the results of Tukey post hoc comparisons). We found no evidence of reduced levels of intact ribosomes in *Spiroplasma*-positive relative to negative flies in *L*. *boulardi*-infested *D*. *melanogaster (*ANOVA: *F*_2,21_ = 4.08, *p* = .032, a modest 1.4-fold increase in intact templates detected in S+ flies) or in *L*. *heterotoma*-infested *D*. *neotestacea (*ANOVA: *F*_2,19_ = 0.36, *p* = .705). *L*. *heterotoma*-infested *D*. *melanogaster* appeared to show a reduction in intact ribosomes (ANOVA: *F*_2,21_ = 13.9, *p* < .001) associated with *Spiroplasma* infection, as well as an apparent increase in detectable intact templates in the *Spiroplasma*-positive, wasp-uninfested treatment (Fig 3A, 3C and 3E). Because this effect is more pronounced in flies succumbing to wasp infestation, i.e. during *L*. *heterotoma* but not *L*. *boulardi* parasitism, it is possible that there is some loss of ribosomal integrity associated with fly mortality in *D*. *melanogaster*, a result we do not observe in *D*. *neotestacea*. Finally, to test for delayed RIP activity on adult hosts, we measured levels of intact and depurinated ribosomes in week-old adult *Spiroplasma*-positive *D*. *melanogaster* that had survived *L*. *boulardi* parasitism, and found no significant change in abundance of depurinated host ribosomes compared to unexposed controls (S1 Fig; t-tests: intact, t_10_ = 0.20, *p* = .849; depurinated, t_10_ = 1.30, *p* = .221).

We tested whether RIPs are encountering host ribosomes intracellularly, or if free host ribosomes are being depurinated in the hemolymph, where *Spiroplasma* resides, which could account for the RIP activity we found in host flies. We bled third instar *D*. *neotestacea* larvae into Ringer’s solution and separated hemolymph from hemocytes (i.e. blood cells) by centrifugation, then assayed cell pellets and supernatant for RIP activity. We found 11-fold greater levels of depurination in the hemolymph than in the cell pellet, and 125-fold greater levels of depurination in hemolymph than in the bled larval carcasses. Levels of depurination were significantly different across RNA isolation sources (Fig 3G, ANOVA: *F*_5,29_ = 32.66, *p* < .001) with levels of depurination in hemolymph, i.e. sourced from extracellular ribosomes, significantly greater than in hemocytes, bled larvae, and control larvae (Tukey test: *p* < .001). The proportion of depurinated ribosomes in the cell pellets, enriched for ribosomes within hemocytes, was also significantly greater than in bled larvae (*p* = .002) and not greater than controls (*p* = .112). Compared with the levels of depurinated host and wasp ribosomes we report from the wasp exposure experiments in *D*. *neotestacea*, the proportion of host ribosome depurination in hemolymph is not significantly different from the highest proportion of wasp depurination we observed but is greater than in wasp-infested host larvae from these experiments (*p* < .001). These results suggest that the low levels of host depurination we see are due to depurination of free host ribosomes that have been released into the hemolymph where there is abundant *Spiroplasma*.

### Diversity of RIPs in the defensive *Spiroplasma* of *D*. *neotestacea*

**Fig 4:**
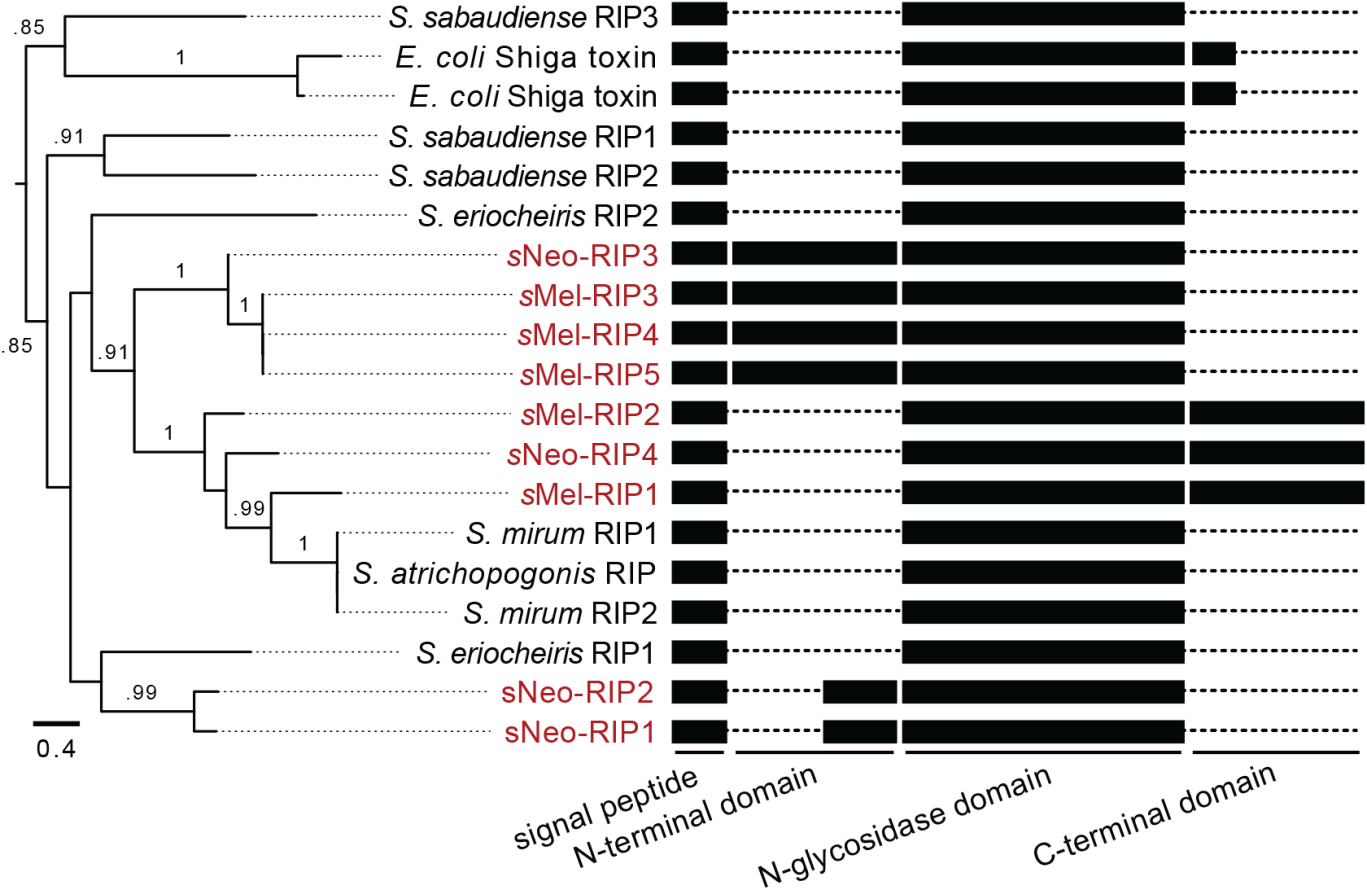
Diversity of ribosome-inactivating proteins in *Spiroplasma*. A maximum likelihood phylogram of *Spiroplasma* ribosome-inactivating proteins (RIPs), along with a diagram showing putative domains. *E*. *coli* Shiga toxin is also included. Sequences from defensive *Spiroplasmas* of *Drosophila* are shown in red. RIP number designations correspond with the numbering system used for RIP transcripts elsewhere in the manuscript. Branches are labeled with approximate likelihood-ratio test scores of .75 or higher.

Previous work focused on a single RIP, *s*Neo-RIP1, that is upregulated in response to nematode exposure in *D*. *neotestacea*, and that depurinates at the α-sarcin/ricin loop of eukaryotic 28S rRNA [32,40]. Interestingly, the *Spiroplasma* from *D*. *melanogaster* encodes five putative RIPs. We predicted therefore that *s*Neo may harbor additional RIP diversity. We generated *s*Neo Illumina and PacBio sequence and screened our draft genome assembly, identifying a total of four putative RIPs. Two are phylogenetically distinct from all of the RIPs of *s*Mel (*s*Neo-RIP1 & 2), while the two others (*s*Neo-RIP3 & 4) have close relatives in *s*Mel (Fig 4). All four of the putative *s*Neo RIPs return significant matches to the ricin A-chain (HMMER e-values < E^-05^ to pfam PF00161). We searched for putative lectin domains (B-chains) within these RIP-coding sequences but failed to identify any candidate domains. We confirmed each new *s*Neo RIP by PCR amplification and Sanger sequencing.

RIPs in *Spiroplasma* from *D*. *melanogaster* and *D*. *neotestacea* represent three distinct groups based on predicted protein domains and phylogenetic analysis (Fig 4). The first clade is composed of *s*Neo-RIPs 1 and 2, which contain a short predicted disordered region between the predicted signal peptide and N-glycosidase domain, as reported for *s*Neo-RIP1 [40]. The second clade contains *s*Neo-RIP3 and *s*Mel-RIPs 3, 4, and 5, and encodes a longer N-terminal domain of ~150 aa, which is not predicted to be disordered by HMMER [44]. The third clade contains *s*Neo-RIP4 and *s*Mel-RIPs 1 and 2, which appear to lack an N-terminal domain, and encode a unique ~150 aa long C-terminal tail. Neither the N- or C-terminal domains produce significant hits via HMMER to indicate putative function of these domains.

We quantified baseline transcript abundance of each *s*Neo and *s*Mel RIP throughout development of unparasitized *D*. *neotestacea* and *D*. *melanogaster*, starting 24 hours after the flies entered the second larval instar stage, and at three more 24-hour intervals. Each RIP transcript was quantified for six biological replicates at each time point. We found that although all *s*Mel and *s*Neo RIPs were constitutively expressed, they differed in abundance (ANOVA: *s*Neo RIPs, *F*_3,92_ = 109, *p* < .001; *s*Mel RIPs, *F*_3,69_ = 20.1, *p* < .001, S2 Fig).

### Little evidence of RIP attack in a parasitic wasp that is resistant to *Spiroplasma*

**Fig 5:**
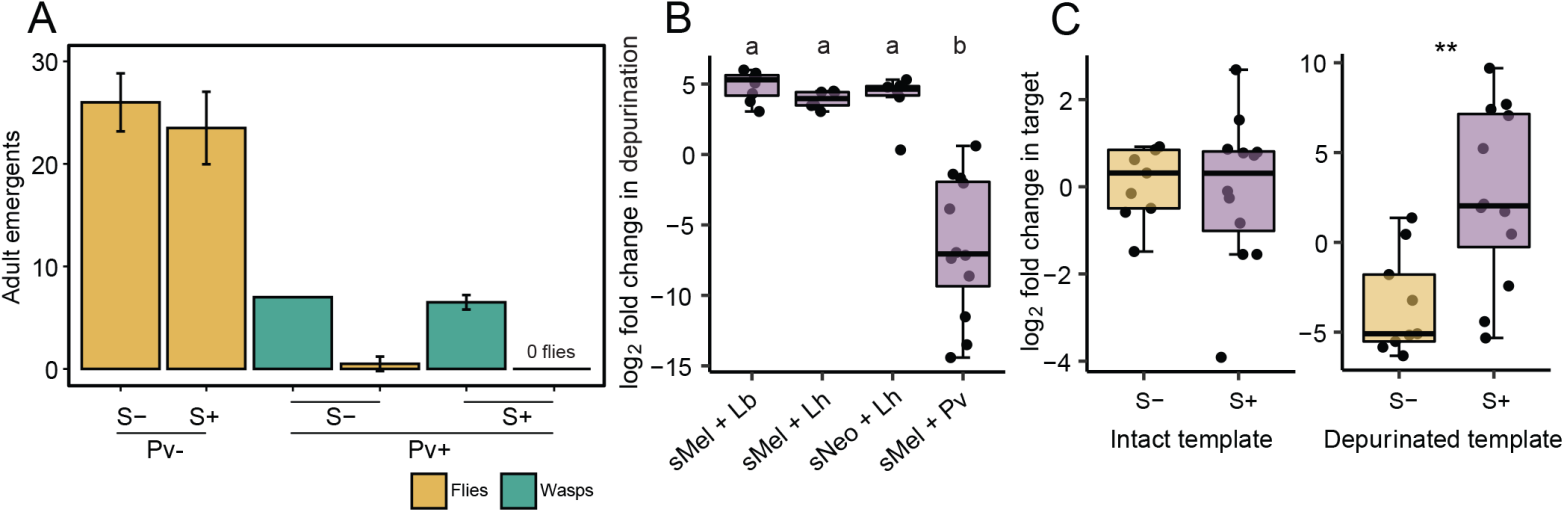
The ectoparasitic wasp *Pachycrepoideus vindemmiae* successfully develops in *Spiroplasma*-positive *Drosophila melanogaster* and does not show evidence of RIP attack. *Pachycrepoideus vindemmiae* (Pv) successfully parasitizes *Spiroplasma*-positive *D*. *melanogaster* (A). Neither fly nor wasp emergence was significantly affected by the presence of *Spiroplasma* (*p* ≥ .5). The proportion of depurinated ribosomes is much less than what is seen in *Leptopilina* species (*p* < .001; B). There is no difference in the level of intact *P*. *vindemmiae* ribosomes in *s*Mel-positive flies compared to levels in *s*Mel-negative flies (*p* = .778). Levels of depurinated ribosomes in *s*Mel-positive flies are modestly, albeit significantly, greater than in *s*Mel-negative flies (*p* = .004; C). Twelve *s*Mel-positive and nine *s*Mel-negative fly pupae were tested for RIP activity and jitter points are the mean of two technical replicates per wasp-infested larva. Significant comparisons from Tukey post hoc tests are labeled above boxplots.

We were interested in examining RIP attack in a wasp that we predicted might overcome protection by *Spiroplasma*. *Pachycrepoideus vindemmiae* (Hymenoptera: Pteromalidae) is a pupal ectoparasitic wasp [45] that pierces the fly pupal case with its ovipositor and deposits an egg on the cuticle of the developing fly inside. Thus the developing wasp larva feeds on but does not develop surrounded by host hemolymph like *Leptopilina*. As we expected, *P*. *vindemmiae* successfully parasitizes *Spiroplasma*-positive flies (Fig 5A). We detected depurinated ribosomes, but much less than for *L*. *heterotoma* and *L*. *boulardi* (Fig 5B, ANOVA: *F*_3,28_ = 27.8, *p* < .001). Depurinated ribosomes were 67 times more abundant in *Spiroplasma*-positive samples than *Spiroplasma-*free controls (t-test: t_19_ = 3.27, *p* = .004), and there was no reduction in intact ribosomes, unlike *Leptopilina* (Fig 5C, Welch’s test: t_16.6_ = 0.29, *p* = .778).

## Discussion

In this paper, we implicate ribosome-inactivating proteins as key players in *Spiroplasma*-mediated protection against parasitic wasps, suggesting that similar mechanisms are used to defend against nematodes and wasps. We show very high levels of depurination at the α-sarcin/ricin loop for wasp ribosomes developing in *Spiroplasma*-positive flies. Depurination occurs very early, soon after the wasp larva hatches. Likewise, we show a marked decrease in levels of intact wasp ribosomes.

Interestingly, we found that the pupal ectoparasitic wasp, *Pachycrepoideus vindemmiae*, is not affected by *Spiroplasma*—nor does it suffer from high levels of ribosome attack. We do detect some depurinated *P*. *vindemmiae* ribosomes, but at significantly lower levels than *L*. *boulardi* and *L*. *heterotoma*. This suggests that RIP effectiveness may depend on symbiont location and titer. Endoparasitic wasp larvae (as well as nematode motherworms) are bathed in *Spiroplasma*-infested hemolymph, and likely experience a much higher degree of exposure to toxins than externally-feeding parasites or gut parasites. Like *Leptopilina*, *Pachycrepoideus* feeds on fly hemolymph, but living outside of the host, it is not immersed in hemolymph upon hatching. Thus it may not be surprising that *Pachycrepoideus* overcomes *Spiroplasma* defense; although, ingesting plant RIPs has been shown to be highly toxic to some insects, such as *Callosobruchus* and *Anthonomus* beetles [46], and *Pachycrepoideus* may be ingesting significant levels of toxin, since *Spiroplasma* titer increases dramatically during fly pupation [25]. Resistance to ricin has been demonstrated in houseflies [47] and grasshoppers [48] and to ricin and saporin (a type 1 RIP) in *Spodoptera* and *Heliothis* moths [46], where reduced RIP toxicity has been attributed to serine protease-mediated hydrolysis in the digestive tract, and it is possible that RIPs are also broken down in *Pachycrepoideus'* gut.

What determines a symbiont's ability to defend against one type of natural enemy and not another, and to avoid harming hosts? A potential clue may be found in the diversity of RIPs encoded by *s*Neo and *s*Mel, which encode 4 and 5 divergent RIPs, respectively, despite having highly reduced genomes typical of vertically transmitted endosymbionts [2,41]. Furthermore, some of these divergent RIPs are not shared between the two symbionts, even though they are both strains of *Spiroplasma poulsonii*. We speculate that the different RIPs are specialized for targeting different cell types. This may explain why *s*Neo but not *s*Mel defends against nematodes [23], as it encodes two divergent RIPs that *s*Mel lacks. It will be interesting to determine whether *Spiroplasma* RIPs respond differentially to different host natural enemies or stresses.

We do not yet know how *Spiroplasma* RIPs target and enter specific host or parasite cells. Although *Spiroplasma* RIPs are type 1 and do not contain lectin domains, some, but not all, have N- or C-terminal domains with no known homologs; perhaps these are important in target specificity. The discovery of signatures of RIP activity in *Spiroplasma*-infected *D*. *melanogaster* opens up the possibility of engineering transgenic flies that express *Spiroplasma* RIPs to directly test these questions, and to confirm a causal role of *Spiroplasma* RIPs in protection. Some type 1 RIPs, such as saporin and trichosanthin in plants, have been shown to require interaction with surface receptors for cell entry [49,50]. For example, in mammalian cells, endocytosis of saporin is mediated by a member of the low density lipoprotein (LDL) receptor family, called LRP1. It was recently proposed that lipids may play an important role in *Spiroplasma* protection against wasps [25], as both *Spiroplasma* and wasps take up lipids from their host [51,52]. In insects, uptake of lipophorin, the major lipid carrier in *Drosophila* hemolymph [53], is mediated by an insect relative of LRP1, VLDL [54]. Thus, it is tempting to speculate that parasitic wasp adaptations to increase lipid uptake, such as elevated production of VLDL receptors, could also result in enhanced vulnerability to RIPs. Recently, Mateos et al. (2016) [55] found that some endoparasitic wasps, such as *Leptopilina guineaensis*, are resistant to *Spiroplasma*, and it would be interesting to see whether resistant wasps differ in VLDL receptor sequence.

Toxins that target highly conserved cellular components may be favored for their utility against a range of natural enemies, but the benefit of this generality may also come at a cost to the host. However, we found little evidence of negative effects of *Spiroplasma* RIPs on hosts. This suggests that mortality of *Spiroplasma*-positive flies attacked by *L*. *heterotoma* (but not *L*. *boulardi*) is not due to the symbiont. Differential fly mortality is probably due instead to differences between *Leptopilina* species, particularly with respect to their venoms, which are very different [56,57]. A recent study found that *L*. *heterotoma* venom is extremely toxic to *Drosophila*, killing flies even when no wasp eggs are deposited [58]. Interestingly, even though there is little evidence of negative effects of wasp or nematode [40] protection on flies, it is worth noting that *Spiroplasma* does kill flies: *s*Mel kills male embryos [26,29,59,60] and also causes pathology in old adult females [51].

We have shown that *Spiroplasma* symbionts encode a diverse assemblage of RIP toxins that appear to play an important role in defense against parasitic wasps, as well as parasitic nematodes. Toxin diversity and expansion may be a common feature in defensive symbionts [11,61,62], and a major outstanding question is how this toxin diversity mediates specificity [63] and coevolutionary arms races between symbionts, hosts, and enemies.

## Materials and Methods

### Host, symbiont and wasp strains

To avoid confusion, we refer to the strains of *Spiroplasma poulsonii* that infect *D*. *melanogaster* and *D*. *neotestacea* as *s*Mel and *s*Neo, respectively. This terminology is similar to how *Wolbachia* strains that infect *Drosophila* are called (i.e. *w*Mel [64]). *s*Mel is also known as *Spiroplasma poulsonii* MSRO (melanogaster sex ratio organism) because of its male-killing phenotype. This strain was originally collected in *D*. *melanogaster* in Uganda, Africa [65] and was introduced into the Oregon-R genetic background by microinjection and provided to us by Bruno Lemaitre’s lab at EPFL. Male-killing in *S*. *poulsonii* MSRO is highly penetrative, such that every generation is 99–100% female. We therefore mated *Spiroplasma*-infected females to uninfected Oregon-R males every generation to maintain the culture. It is important to note that because of this penetrance, all larvae of the *Spiroplasma*-positive treatment of *D*. *melanogaster* are female, different to the *Spiroplasma*-free treatment and *D*. *neotestacea* which have unbiased sex ratios (*s*Neo does not kill males). *D*.*neotestacea* and its *Spiroplasma* symbiont (*s*Neo) were collected in West Hartford, CT, USA in 2006. Fly lines are maintained on Instant *Drosophila* Medium (Carolina Biological Supply) on a 12:12 light dark cycle, *D*. *melanogaster* at 24°C and *D*. *neotestacea* lines at 21°C with the addition of a cotton dental roll and a small slice of *Agaricus bisporus* mushroom for *D*. *neotestace*a. *L*. *heterotoma* (strain Lh14) is a generalist endoparasitic wasp that successfully parasitizes many species of *Drosophila* and *L*. *boulardi* (Lb17) is a specialist endoparasitic wasp of *Drosophila melanogaster* and other members of the melanogaster group [66]. These wasp strains were initially collected by Todd Schlenke in Winters, California in 2002 and were provided by the Lemaitre lab and maintained on second instar Oregon-R larvae at 24°C in a vial with a moistened dental cotton roll and white sugar. *Pachycrepoideus vindemmiae* is a generalist pupal ectoparasitic wasp of many cyclorrhaphous flies [67] and was provided by Dr. Joan Cossentine (Agriculture and Agri-Food Canada) and maintained on Oregon-R pupae at 24°C in vials with a dental cotton roll that had been dipped in a 10% white sugar solution.

### Wasp exposures

To ensure highly efficient vertical transmission of *Spiroplasma*, parental flies were aged at least five days prior to egg laying. Adults were placed on fresh instant food or mushroom vials for 24 hours then removed. From these vials, three-day-old second-instar larvae were picked and placed on instant food in 35 mm petri dishes. We found that *D*. *neotestacea* pupal survival was negatively affected by picking and transplanting them to dishes, often because they preferred to crawl into the narrow lid opening to pupate. For this species we carried out wasp exposures in vials with a small piece of mushroom such that larvae could not burrow deep enough to avoid wasp ovipositors. *Leptopilina* exposures were done in duplicate or greater with at least 40 fly larvae per dish. Five mated female wasps with experience parasitizing Oregon-R were added to each dish and allowed to oviposit for 48 hours. *Pachycrepoideus* exposures were also done in duplicate petri dishes with 30 two-day-old fly pupae presented to five experienced female wasps for 48 hours. Wasp success in oviposition was inferred from detection of wasp rRNA by RT-qPCR and was 100% for *L*. *heterotoma* and *L*. *boulardi* on *D*. *melanogaster*, 69% for *L*. *heterotoma* on *D*. *neotestacea*, and in 62% for *P*. *vindemmiae* on *D*. *melanogaster*.

For timecourse experiments, the *Leptopilina* protocol above was altered to shorten *Drosophila* egg-laying time to eight hours as well as wasp exposure time to three hours in an effort to better synchronize both fly and wasp development across samples. For the *L*. *heterotoma* timecourse in *D*. *melanogaster*, 30 second-instar fly larvae were moved into a 35 mm petri dish, as described above, for each of two replicate dishes per treatment. Despite briefer exposures, parasitism rates were high; wasp rRNA was detected in 69 of 72 wasp-exposed flies.

We performed an additional exposure of *D*. *melanogaster* to *L*. *heterotoma* in order to determine the timeframe of wasp hatching within *Spiroplasma*-positive fly larvae. As done previously, 40 second-instar larvae were exposed to five experienced *L*. *heterotoma* females for three hours in a dish, afterward the wasps were removed. 48 h after the start of wasp exposure, 20 larvae were removed and dissected under a Leica MZ6 dissecting light microscope and each was visually scored for the presence of a hatched or unhatched wasp. At 72 h post-exposure, dissections were again carried out, on all of the remaining live larvae (n = 11), and larvae were scored for hatched or unhatched wasps.

### RNA extractions and RT-qPCR

Following wasp exposures, *Drosophila* pupae were collected for RNA extraction. For *Leptopilina* exposures, we collected first- and second-day-old pupae, four pupae from each of two replicate dishes or vials per treatment. For the *L*. *heterotoma* in *D*. *melanogaster* timecourse, three larvae or pupae were collected from each replicate dish (six per treatment) at the each of the following time points post-exposure: 3 h, 24 h, 48 h, 72 h, 96 h, and 120 h. Flies were larvae at the first four collection points, and were pupae at 96 h and 120 h.

RNA was extracted from all samples in the same way regardless of fly species or age: Flies were homogenized for 10–15 seconds with a bead-beater in a 1.5 mL microfuge tube with 300 uL TRIzol reagent (Invitrogen) and approximately ten 1 mm zirconia beads (BioSpec Products). TRIzol extractions were carried out according to the manufacturer’s recommended protocol. RNA was quantified on a NanoDrop spectrophotometer. 1 μg or, for some of the smallest larvae extracted, 0.5 μg, was used for cDNA synthesis immediately following quantification. cDNA synthesis reactions used SuperScript II reverse transcriptase (Invitrogen) and were each primed with 50 ng of random hexamer primers (Integrated DNA Technologies). RT-qPCR reactions were done in duplicate with SYBR Select Master Mix (Applied Biosystems) in a Biorad C1000 Touch Thermal Cycler with a CFX96 Real-Time System interfaced with CFX Manager 3.0 software. Ct values across replicates did not vary by more than 0.5 C_t_. For timecourse reactions, all reactions for one primer set could not be amplified in a single plate so an interplate calibrator of pooled samples was used to normalize samples. Primers used throughout this study and the details of their validation are provided in S1 Table.

### Validation and analysis of RIP activity assays

RIPs are N-glycosidases that target an adenine residue in the α-sarcin/ricin loop of eukaryotic 28S rRNA. We used an established RT-qPCR based assay to quantify RIP activity [40,42,43]. In brief, the N-glycosidase activity of RIPs leaves an abasic site at this adenine, and when reverse transcriptase encounters this position during cDNA synthesis, it incorporates a deoxyadenosine monophosphate (dAMP) into the nascent strand, while complementary DNA (cDNA) constructed from intact templates will receive a thymidine monophosphate (TMP). For detection of these targets by RT-qPCR, forward primers were designed such that the 3’-most primer position complements intact or depurinated variants of the RIP-targeted position, and a secondary mismatch was introduced at an adjacent position for both primer sets to improve specificity. The reverse primer targets a nearby region of 28S rRNA exhibiting high sequence divergence between *Leptopilina/Pachycrepoideus* and *Drosophila*. Primer sequences and further details of their validation are presented in S1 Table. We tested the specificity of this assay on IDT gBlocks synthetic DNA with either A or T residues at the RIP-targeted site. We found it to be highly-sensitive to detect this single nucleotide base change, i.e. cross-template amplification by our primers does not occur until 17–20 C_t_ (cycle threshold) values after the intended target, meaning fold-changes of 130,000–1,000,000 are the upper limit of detection for this assay. A product for each of the RT-qPCR targets was Sanger sequenced to confirm product identity and following each reaction, melt curves were examined to confirm that melting temperature matched the expectation for each product.

Fold changes were calculated by the Pfaffl ratio method [68]. Here ΔCt is calculated by subtracting the C_t_ of each treated sample from an untreated sample. We used the global mean Ct for each primer set in place of the untreated sample which facilitates the plotting of untreated (S-) data. Primer efficiency (E) was incorporated as 1+E^ΔCt^ for each primer and ratio of gene of interest to reference was calculated as usual. The reference target used to normalize intact and depurinated targets was a nearby region of the 28S rRNA. The reference gene used to normalize RIP transcript abundance was the *Spiroplasma* DNA-directed RNA polymerase subunit B (*rpoB*). Data were graphed in R version 3.3.3 using the ggplot2 package.

To quantify relative changes in RIP transcript abundance, we performed RT-qPCR on cDNA samples generated from unparasitized host larvae during time course experiments. We normalized RIP expression in each of six biological replicates per time point to the corresponding *rpoB* reference transcript data and calculated –ΔCt values to compare RIP transcript abundances to one another. We analyzed the results with ANOVAs to test for significant effects of time and RIP copy. We note that in *s*Mel, RIPs 3,4, and 5 are nearly identical at the nucleotide level, probably the result of recent gene duplication. As a result, our primers do not differentiate between their transcripts and we refer to them collectively as *s*Mel RIPs3-5.

### Hemolymph RIP assay

To test whether fly hemolymph is enriched for depurinated host ribosomes, third instar *D*. *neotestacea* larvae were rinsed in Ringer’s solution and anesthetized on ice. Eight larvae were bled into 500 μL of ice cold Ringer’s solution for ten minutes after piercing the cuticle with a needle. This was done in separate 35 mm petri dishes to generate biological replicate pools. Larval carcasses were collected and stored in Ringer’s solution on ice while hemolymph solutions were centrifuged for 10 minutes at 3,000 rpm at 4°C to pellet hemocytes. Pellets were observed in each tube and the supernatant (hemolymph) fraction was transferred to a separate tube. The pellet was rinsed with cold Ringer’s solution and centrifuged briefly to remove residual supernatant in the tube. RNA was extracted from all fractions as well as pools of unbled control larvae using TRIzol. cDNA synthesis was done using 100 ng of input RNA per sample and RIP assays were carried out as described previously. This experiment was done once initially with two biological replicate pools per tissue type and repeated with four more biological replicate pools per tissue type.

### Identification and phylogenetic analysis of ribosome-inactivating protein genes in *s*Neo

Because *s*Neo does not grow in culture, we prepared *s*Neo genomic DNA extractions from host tissues enriched for *Spiroplasma*. We dissected ovaries from approximately 40 three-week-old *D*. *neotestacea* flies and extracted DNA using the phenol-chloroform method. We sequenced the *s*Neo genome using Pacific Biosciences RSII and Illumina HiSeq 2500 sequencing technologies (Genome Quebec). We sequenced long reads on three SMRT cells and short reads in 0.4 of a sequencing lane. A preliminary *s*Neo genome was assembled by Genome Quebec using long reads only via the SMRT Pipeline, with an estimated genome size of 1.8 Mb. The assembly contains *Drosophila*-derived sequences, decontaminating and error-correcting these data is an ongoing project. We searched for RIP genes within this preliminary assembly by tblastn using the *s*Mel RIPs and *s*Neo-RIP1 as query sequences and confirmed the presence and sequence of each with PCR and Sanger sequencing.

Phylogenetic analysis was performed on *Spiroplasma* RIP sequences from *s*Neo, *s*Mel, and other sequenced *Spiroplasma* genomes, which were identified by blastp and tblastn against GenBank’s non-redundant protein and genomic sequence databases. *Spiroplasma* matches with expected value less than 10^−5^ were retained. Amino acid sequences were aligned by MAFFT under the E-INS-i alignment algorithm. The putative domain divisions shown in Fig 4 were determined from this alignment. The best protein substitution model was selected using ProtTest 2.4 [69]. The selected model under Bayesian information criterion was WAG+I+G+F. A phylogram was constructed and SH-like approximate likelihood-ratio scores calculated with PhyML 3.0 [70] implemented by SeaView 4.5.4 [71].

## Supporting information

S1 Fig No evidence of delayed RIP activity due to a previous wasp exposure in surviving adult *Drosophila*. Levels of intact and depurinated ribosomes in one-week-old *Spiroplasma*-positive adult flies remain unchanged between flies that survived wasp defense (blue) and those that were not exposed to a wasp (pink; intact *p* = .849; depurinated *p* = .221). Jitter points are the mean of two technical replicates per larva. Significant comparisons from Tukey post hoc tests are labeled above boxplots.
(PDF)

S2 Fig Transcript abundance of RIPs during larval development. Results of RIP transcript abundance changes as measured by RT-qPCR across *Spiroplasma*-infected host development in two species of *Drosophila*, beginning 24 hours after larvae reached the second larval instar stage. Each RIP was measured in six larvae per time point and its expression was normalized to the *Spiroplasma* DNA-directed RNA polymerase subunit B (*rpoB*). Relative expression levels, or the change in qPCR cycle threshold values (-ΔCt) are plotted for *s*Neo RIPs 1–4 (A) and *s*Mel RIPs 1,2, and 3–5 (C). Also plotted are relative transcript abundances for each RIP pooled across time points for *s*Neo (B) and *s*Mel (D). Results of post hoc significance testing, when significant, are indicated above boxplots (Tukey tests).
(PDF)

S1 Table Validation details and sequences of nucleotide primers used in this study. Primers were validated using cDNA generated with random hexamer oligos as described in the materials and methods. Standard curves were produced from cDNA samples in a series of five 10-fold serial dilutions and each reaction was run as three technical replicates. Efficiency values and R^2^ statistics were calculated by the Bio-Rad CFX Manager 3.0 software.
(PDF)
