# Correction: Generality of toxins in defensive symbiosis: Ribosome-inactivating proteins and defense against parasitic wasps in *Drosophila*

**DOI:** 10.1371/journal.ppat.1013193

**Published:** 2025-05-20

**Authors:** Matthew J. Ballinger, Steve J. Perlman

The hyperlink in the Data Availability statement is incorrect. The correct hyperlink is: https://datadryad.org/dataset/doi:10.5061/dryad.7jf10
