# Supplementary figures and images for: Generality of toxins in defensive symbiosis: Ribosome-inactivating proteins and defense against parasitic wasps in Drosophila
Source: PLoS Pathog. 2017 Jul 6;13(7):e1006431. doi: 10.1371/journal.ppat.1006431 (PMC5500355; doi:10.1371/journal.ppat.1006431)

*D. melanogaster* adults (1 week old)

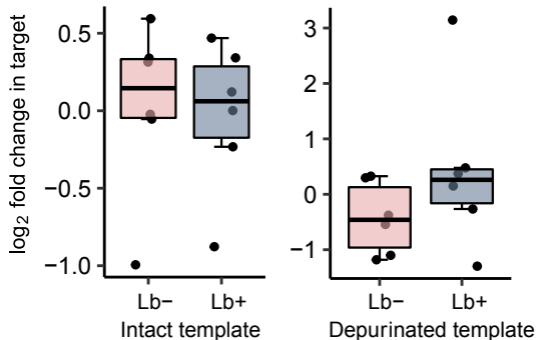

Supplement: S1 Fig — Levels of intact and depurinated ribosomes in one-week-old Spiroplasma-positive adult flies remain unchanged between flies that survived wasp defense (blue) and those that were not exposed to a wasp (pink; intact p = .849; depurinated p = .221). Jitter points are the mean of two technical replicates per larva. Significant comparisons from Tukey post hoc tests are labeled above boxplots. (PDF) [file ppat.1006431.s001.pdf]

A

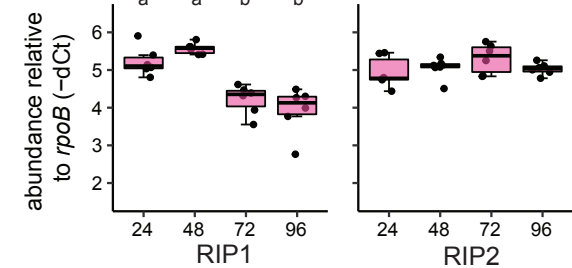

B

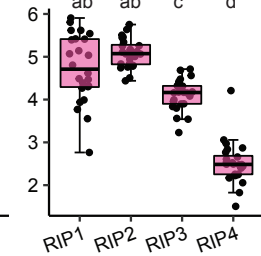

C

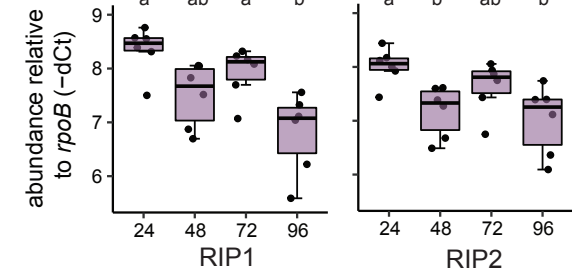

D

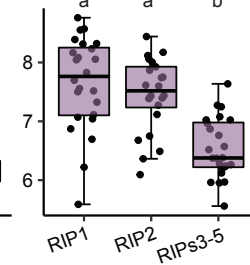

Supplement: S2 Fig — Results of RIP transcript abundance changes as measured by RT-qPCR across Spiroplasma-infected host development in two species of Drosophila, beginning 24 hours after larvae reached the second larval instar stage. Each RIP was measured in six larvae per time point and its expression was normalized to the Spiroplasma DNA-directed RNA polymerase subunit B (rpoB). Relative expression levels, or the change in qPCR cycle threshold values (-ΔCt) are plotted for sNeo RIPs 1–4 (A) and sMel RIPs 1,2, and 3–5 (C). Also plotted are relative transcript abundances for each RIP pooled across time points for sNeo (B) and sMel (D). Results of post hoc significance testing, when significant, are indicated above boxplots (Tukey tests). (PDF) [file ppat.1006431.s002.pdf]
