# Supplementary material for: Generality of toxins in defensive symbiosis: Ribosome-inactivating proteins and defense against parasitic wasps in Drosophila
Source: PLoS Pathog. 2017 Jul 6;13(7):e1006431. doi: 10.1371/journal.ppat.1006431 (PMC5500355; doi:10.1371/journal.ppat.1006431)
Supplement: S1 Table — Primers were validated using cDNA generated with random hexamer oligos as described in the materials and methods. Standard curves were produced from cDNA samples in a series of five 10-fold serial dilutions and each reaction was run as three technical replicates. Efficiency values and R2 statistics were calculated by the Bio-Rad CFX Manager 3.0 software. (PDF) [file ppat.1006431.s003.pdf]

S1 Table. Validation details and sequences of nucleotide primers used in this study

Primers were validated using cDNA generated with random hexamer oligos as described in the materials and methods. Standard curves were produced from cDNA samples in a series of five 10-fold serial dilutions and each reaction was run as three technical replicates. Efficiency values and R^2 statistics were calculated by the Bio-Rad CFX Manager 3.0 software.

Primer name	Primer Sequence	Efficiency	R^2	Application note
sNeoRIP1_f	ATTGTGGAAGAAACAAAACACC	104.6	0.995	
sNeoRIP1_r	TAATGCCATCGACATATAAATTGC			
sNeoRIP2_f	AACAACCAAAAGTGCTAGAGAAAG	103.8	0.999	
sNeoRIP2_r	GGGTGTGTTGTTTCTTCTACAATAT			
sNeoRIP3_f	CAGAATGAACGGCACTTACC	100.4	0.99	
sNeoRIP3_r	TGTTCCATGAGCACTATTTTGTC			
sNeoRIP4_f	CTAGAAGTTTTGGATTCTCTGGTAA	96.9	0.997	
sNeoRIP4_r	TACTGAAACATTACCTGAAGAACTTGG			
msroRIP1_f	TGGACTTCGAGCTGATGAAA	92.1	0.99	GenBank accession JTLV01000005
msroRIP1_r	GCTGTTGCCAAAATAACACG			
msroRIP2_f	CATGGTATTTCAGAGCACTATGC	104.4	0.991	GenBank accession JTLV01000006
msroRIP2_r	TGTATCATTACCTCAACTAACCTTAG			
msroRIP3-5_f	TAATAACTAGACCATTAGTTAGACGAA	104.1	0.994	GenBank accessions JTLV01000002, JTLV01000008,
msroRIP3-5_r	CTGCCAAATTGTTCTATTATTCCTTCT			JTLV01000009
rpoB_f	GTTTTTGATGGAATGACAAATGAAGAATT	99.8	0.998	
rpoB_r	CGAAACTGGATTATCAAATTTTTCACC			
wasp_f_cut	TAGTAATCCTGCTCAGTATGT	107.9	0.998	pair with wasp r to target depurinated wasp SRL
wasp_f_uncut	TAGTAATCCTGCTCAGTATGA	107.9	0.998	pair with wasp r to target intact wasp SRL
wasp_r	TCGTTGCCTCCTTTGACTAGA			
Lepto_28S_f	CAATTCGGGCTCTCGCAAGA	111.3	0.997	*Leptopilina* ribosomal reference
Lepto_28S_r	GTGCTCTTCGCGTTCCAA			
Pachy_28S_f	CCTCGTAAGAGTGTTCGTCG	98.7	0.998	*Pachycrepoideus* ribosomal reference
Pachy_28S_r	GTGCTCTTCGCGTTCCAA			
fly_f_cut	CGACAGCATTCCTGCGTAGTAAGT	102.4	1	pair with fly_r to target depurinated fly SRL
fly_f_uncut	CGACAGCATTCCTGCGTAGTAAGA	106.5	0.999	pair with fly_r to target intact fly SRL
fly_r	ACAATGCAAATTGCCCCTTA			
Dros_28S_f	GTTCCAATTCCGTAACCTGTTGAG	103.4	0.997	*Drosophila* ribosomal reference
Dros_28S_r	GTCATGCTCTTCTAGCCCATCT			
